# Multifocal Ocular Manifestations Heralding Relapse of Acute Myeloid Leukemia: A Case Report and Literature Review

**DOI:** 10.3390/jcm14217506

**Published:** 2025-10-23

**Authors:** Elvia Mastrogiuseppe, Maria Carmela Saturno, Clara Minotti, Martina Angi, Marco Marenco

**Affiliations:** 1 Department of Sense Organs, Medicine and Dentistry Faculty, Sapienza University of Rome, Viale del Policlinico 155, 00161 Rome, Italy; mariacarmela.saturno@uniroma1.it (M.C.S.); marco.marenco@uniroma1.it (M.M.); 2 Department of Translational and Precision Medicine, Sapienza University of Rome, Via Benevento 6, 00161 Rome, Italy; clara.minotti@uniroma1.it; 3 Ocular Oncology Unit, Department of Surgical Oncology, Fondazione IRCCS Istituto Nazionale dei Tumori, Via Giacomo Venezian, 1, 20133 Milan, Italy; martina.angi@istitutotumori.mi.it

**Keywords:** acute myeloid leukemia, ocular involvement, choroidal infiltration, orbital proptosis, leukemic retinopathy, multifocal ocular leukemic involvement, sanctuary site, relapse

## Abstract

This case-based review examines the spectrum of leukemic ocular involvement, focusing on its prognostic implications. A rare case of relapsed acute myeloid leukemia (AML) in a 63-year-old man is presented, featuring simultaneous orbital proptosis, adnexal involvement, choroidal and retinal infiltration, and hemorrhagic changes affecting both the anterior and posterior segments. This constellation of findings, affecting multiple ocular structures concurrently, highlights the eye’s potential role as a sanctuary site for leukemic cells and underscores the diagnostic challenge of distinguishing direct infiltration from treatment-related or secondary vascular damage. This case, integrated with a literature review, emphasizes that multifocal ocular signs may serve as early indicators of leukemic relapse and reinforce the need for close collaboration between ophthalmologists and hematologists in guiding patient management.

## 1. Introduction

Acute myeloid leukemia (AML) is an aggressive myeloid malignancy in which immature blasts crowd the bone marrow, disrupting normal hematopoiesis and causing life-threatening pancytopenia [1].

Although classically a systemic disease, AML can infiltrate every ocular compartment, from adnexa and orbital tissues to choroid and optic nerve [2], far more often than its chronic counterparts [3,4]. Ocular findings may precede the hematologic diagnosis or herald a covert relapse, but their presence portends poorer prognosis, being linked to higher rates of medullary relapse or central nervous system (CNS) involvement [5,6].

We describe a rare case of AML relapse manifesting with simultaneous orbital, adnexal, choroidal, and retinal infiltration, accompanied by hemorrhagic involvement of both the anterior and posterior segments. This constellation of findings, affecting multiple ocular structures concurrently, is exceptionally uncommon in adults and strongly suggestive of systemic recurrence. In addition to presenting this case, we review the literature on ocular involvement in leukemia, highlighting its pathogenetic mechanisms and prognostic implications.

## 2. Case Presentation

A 63-year-old man diagnosed with AML FLT3 ITD+, with intermediate risk according to the 2022 European LeukemiaNet (ELN) recommendations [7], underwent consolidation chemotherapy with high-dose Cytarabine (1500 mg/m^2^ every 12 h on days +1, +3, and +5) and Midostaurin (50 mg twice daily), according to the institutional consolidation protocol at Policlinico Umberto I, Rome, Italy. On day 14 of the maintenance regimen, the patient experienced a transient fever spike of 38 °C that led to treatment discontinuation. The patient underwent a CT of the chest and head, which showed no focal abnormalities suspicious for infectious foci. Blood cultures showed no evidence of active infection, but broad-spectrum antimicrobial therapy was nevertheless initiated. Bone marrow aspiration subsequently confirmed aplasia.

**Figure 1:**
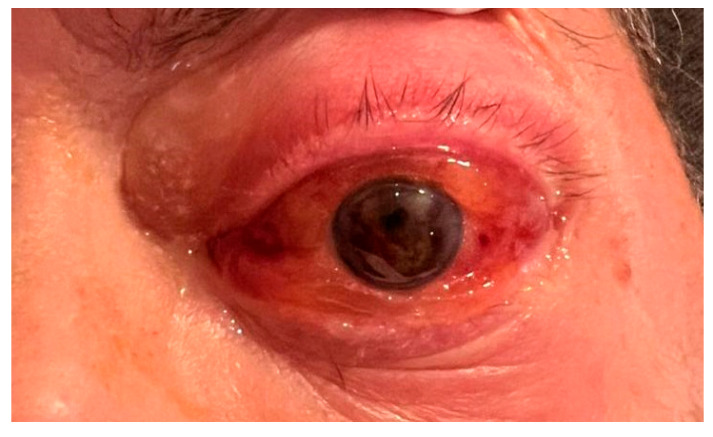
Clinical presentation of leukemic ocular involvement: proptosis and periocular edema, conjunctival chemosis, hyphema, pupil seclusion with epipupillary membrane. These findings reflect anterior segment and adnexal involvement consistent with leukemic infiltration.

Two weeks later, the patient presented with proptosis of the left eye (LE) and blurred vision. No prior ophthalmic records were available; the patient reported a history of good vision without ocular complaints. Bedside ophthalmic examination revealed light perception visual acuity, significant proptosis, periocular edema, conjunctival chemosis and diffuse subconjunctival hemorrhage, hyphema, pupil seclusion with epipupillary membrane in the LE (Figure 1). Indirect ophthalmoscopy could not be performed due to vitreous hemorrhage. The right eye (RE) was unremarkable for pathological signs.

MRI of the brain and orbits was obtained. Neuroimaging demonstrated minute, focal hypointense lesions in both supratentorial and infratentorial regions, compatible with microbleeds, without signal abnormalities suggestive of CNS leukemic localization.

**Figure 2:**
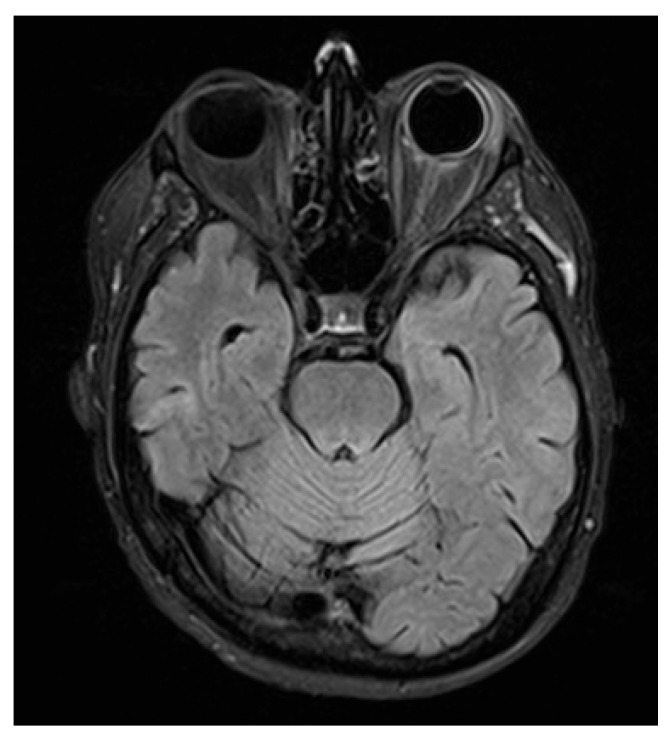
Orbital MRI (T2-FLAIR) showing hyperintensity of the retro- and periorbital fat, posterior sclera, and optic nerve sheath, consistent with leukemic infiltration extending to adnexal and retrobulbar tissues.

**Figure 3:**
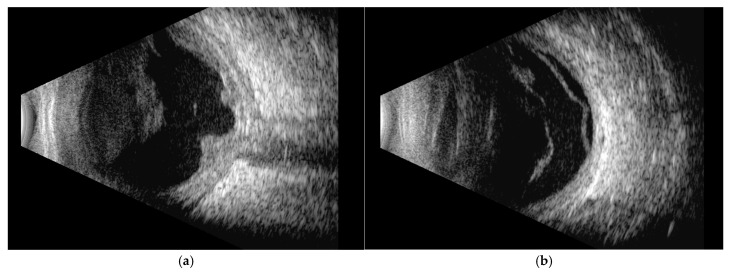
B-scan ultrasonography showing: (**a**) fibrotic consolidations and organized vitreous hemorrhage; choroidal thickening, alteration of the normal contour of the optic disc head, tram-track sign of the optic nerve sheath and hypoechogenicity of the retroscleral tissue. These findings are suggestive of optic nerve sheath and choroidal infiltration rather than hemorrhagic changes; (**b**) exudative retinal detachment.

Orbital T2-FLAIR MRI sequences showed mild edema and hyperintensity of the retroorbital and periorbital fat, the lacrimal gland, the posterior sclera, and the optic nerve sheath (Figure 2). B-scan ultrasonography showed fibrotic consolidations and organized vitreous hemorrhage, along with marked choroidal thickening displaying medium–low reflectivity and elevation of the retinal contour, suggestive of infiltrative material (Figure 3). The associated optic disc distortion and perineural sheath edema, together with MRI findings, supported the diagnosis of multifocal leukemic infiltration with adnexal and retrobulbar extension, rather than hemorrhagic changes.

The hematological evaluation, including a complete blood count, showed hemoglobin of 8.9 g/dL, white blood cell count of 1010/mm^3^, neutrophil count of 430/mm^3^, and platelet count of 15,000/mm^3^. Due to these hematological abnormalities, periocular or transcleral tissue biopsy was contraindicated.

Unfortunately, the patient’s condition continued to deteriorate, and four weeks later, he died from a hemorrhagic stroke, most likely related to bleeding diathesis secondary to chemotherapy-induced bone marrow aplasia, rather than a direct leukemic manifestation.

## 3. Literature Review

### 3.1. Leukemic Ocular Manifestations

The ophthalmic manifestations of leukemia can be classified into two main categories: primary (direct) involvement, due to infiltration of neoplastic cells such as leukemic infiltrates; and secondary (indirect) involvement, from dysregulated hematopoiesis or chemotherapy, causing cytopenias (anemia, thrombocytopenia), hyperviscosity, and immunosuppression with opportunistic infections [8]. These ocular findings occur across acute and chronic leukemias, both myeloid and lymphoblastic. Because they arise at different disease stages and via distinct mechanisms, we will discuss them separately.

#### 3.1.1. Retina

Historically, fundus abnormalities were the most frequently reported ocular manifestation of leukemia [8]; recent comparative and cohort analyses confirm that posterior segment involvement remains common, occurring in up to 64% of cases [9,10,11].

##### Primary Involvement

***Vessel sheathing***

Among the earliest findings are tortuous, dilated retinal veins with a “sausage-like” configuration and vascular sheathing, likely from perivascular leukemic infiltration. Hard exudates and cotton-wool spots may also occur, reflecting nerve fiber layer infarcts or focal leukemic deposits [8,9,12].

***Retinal leukemic infiltrates***

Retinal leukemic infiltrates present as nodular cell accumulations of variable size, often with tissue destruction, necrosis, and hemorrhage; they are generally confined by the internal limiting membrane, though rare vitreous extension, likely from the optic nerve head, has been reported [8,13,14,15]. Large deposits can cause total retinal detachment, sometimes as an isolated relapse [16], whereas smaller infiltrates are typically perivascular [15]. Subretinal infiltration may occur, occasionally appearing as a subretinal hypopyon [17]. Cotton-wool spots may accompany these findings, reflecting ischemia from anemia, hyperviscosity or direct leukemic involvement [13]. Notably, nodular infiltrates correlate with marked leukocytosis with blast predominance and fulminant disease with poor survival; thus, retinal infiltration plus leukocytosis is a negative prognostic marker [8].

##### Secondary Involvement

***Leukemic retinopathy***

The term “leukemic retinopathy” generally refers to retinal changes secondary to anemia, thrombocytopenia, and hyperviscosity, rather than direct leukemic infiltration [18]. It is the most common ocular manifestation, characterized by vessel tortuosity and retinal hemorrhages, typically intraretinal, round or flame-shaped, at the posterior pole. Subhyaloid “boat-shaped” and subretinal hemorrhages are less common; subhyaloid bleeds may rarely break into the vitreous [9,11,19]. It is often seen at relapse, but not pathognomonic nor clearly prognostic and primarily related to severe anemia [8]. In acute lymphoblastic leukemia (ALL), intraretinal hemorrhages correlate with thrombocytopenia and low hematocrit, whereas leukocyte count showed no association; profound anemia plus thrombocytopenia increases risk, implicating hematocrit as a key determinant [20,21,22]. Changes are predominantly reversible, with OCTA showing improved both macular and peripapillary perfusion and increased vessel density in remission [23,24].

***Roth spots***

Roth spots are intraretinal hemorrhages characterized by a central white focus, which may represent cellular debris, capillary emboli, or leukemic aggregates [8]. In acute leukemia they reflect capillary rupture with fibrin–platelet thrombi; proposed drivers include abnormal hematopoiesis, elevated intravascular pressure, ischemia, and increased capillary fragility, leading to endothelial injury and thrombosis [8]. Patients with Roth spots show higher platelet counts than controls [10]; in AML, Roth spots associate with marked leukocytosis or anemia [20]. They are largely reversible with hematologic normalization after chemotherapy or transplantation, with VA improvement despite possible residual macular thinning; recovery varies by disease status and age [10].

***Retinal microaneurysms, capillary closure and neovascularization***

Peripheral microaneurysms occur in half of eyes in chronic leukemia and are uncommon in acute leukemia; they are more frequent in chronic myelogenous leukemia (CML) than chronic lymphocytic leukemia (CLL) [8,11,25]. Prolonged leukocytosis can contribute, but findings are inconsistent [26]. The prevailing mechanism is hyperviscosity from markedly increased leukocytes or platelets, causing flow impairment, peripheral capillary dropout, microaneurysm formation and, rarely, proliferative retinopathy resembling sickle-cell disease [8,13].

***Central retinal vein occlusion (CRVO)***

CRVO is an uncommon leukemic complication, usually linked to hyperviscosity, but it may also result from leukemic infiltration. Infiltration-related occlusions can present with or without disc edema or infiltrates and may even herald CNS involvement (e.g., relapsed AML). Accordingly, retinal vessels occlusions in leukemia warrant evaluation for CNS disease because of prognostic and therapeutic implications [2].

***Opportunistic infections***

Neutropenia (disease- or therapy-related) predisposes to opportunistic ocular infections of viral (CMV, HSV, VZV), fungal (*Candida*, *Aspergillus*), and bacterial origin. Clinical syndromes include necrotizing retinitis, keratitis, uveitis, and endophthalmitis; Candida retinitis may extend into the vitreous with “cotton-ball” opacities, although its incidence has declined with earlier candidemia detection and prompt antifungal therapy [13].

#### 3.1.2. Choroid

##### Primary Involvement

Primary choroidal involvement is clinically uncommon, yet histopathology shows choroidal infiltration in up to 65% of patients and in one-third of post-mortem eyes, making the choroid the most frequently affected ocular site, often at relapse and with concurrent CNS or systemic recurrence [19,27,28]. Symptomatic cases usually show posterior serous retinal detachment in AML, driven by reduced choroidal perfusion, RPE ischemia, barrier dysfunction, and fluid-pump failure, with resolution after systemic chemotherapy [10,13,29,30,31,32]. Choroidal disease may also precipitate angle-closure glaucoma [31,33]. Only two cases have described choroidal infiltration as the first sign of relapse without CNS or systemic disease [27,34].

##### Secondary Involvement

Indirect choroidal involvement is rare. Retinochoroidal infarction has occasionally been observed during ALL therapy [35].

#### 3.1.3. Optic Nerve

##### Primary Involvement

Primary optic nerve involvement in leukemia most often presents with papilledema, typically in association with CNS disease (reported in 13–18%), while histopathology demonstrates optic nerve infiltration in up to one-third of ocular specimens, predominantly in acute leukemia [13,36,37]. Two clinical patterns are described. Prelaminar infiltration appears as superficial, fluffy over the lamina cribrosa, with edema and hemorrhage; vision is often preserved unless there is macular extension. Retrolaminar disease presents as marked disc swelling and hemorrhage with severe vision loss [8,9]. Optic nerve disease is frequent during ALL, may herald relapse, and portends poor prognosis, especially when occurring during active treatment [38,39,40]. Management is challenging because the nerve can serve as a sanctuary site; therapy typically combines intrathecal chemotherapy and radiotherapy [38,41].

##### Secondary Involvement

Chemotherapy, antibiotic or radiotherapy toxicity, ischemia due to anemia or hyperviscosity, and opportunistic infections in immunocompromised patients are among the main causes of secondary optic nerve involvement [39,42,43].

#### 3.1.4. Anterior Segment

##### Primary Involvement

Conjunctival disease may present with hyperemia and edema of the inferior tarsal conjunctiva, occasionally an early sign in ALL, and with perivascular infiltrates or mass-like lesions; rare subconjunctival tumors have been reported in both AML and ALL [8,13,44,45,46]. Corneal infiltration is exceptionally uncommon given its avascularity, but when present it may appear as ring ulcers, subepithelial limbal infiltrates, or peripheral ulceration [8,13,47]. Anterior chamber and iris disease may manifest as uveitis, pseudohypopyon, or spontaneous hyphema, most often in relapsed ALL, with occasional iris infiltration causing heterochromia, hypopyon, and secondary glaucoma; slit-lamp evaluation and, when indicated, anterior chamber paracentesis are critical because ocular or CNS involvement in leukemia signals poor prognosis and may necessitate radiotherapy [8,13,48]. Scleral involvement in ALL often localizes around episcleral vessels, is frequently asymptomatic, and has been detected at autopsy; adult T-cell leukemia can mimic scleritis or episcleritis [49,50].

##### Secondary Involvement

Conjunctival changes due to blood disorders are uncommon, though hyperviscosity in chronic leukemias can produce comma-shaped veins [51]. The most frequent conjunctival pathology is related to graft-versus-host disease (GVHD) after allogeneic transplantation for ALL, typically severe dry eye and keratoconjunctivitis sicca with adverse prognostic implications; chronic GVHD is linked to meibomian gland dysfunction, fibrotic tarsal changes, eyelid malposition, progressive keratinization, and recurrent corneal erosions or ulcers [13,52]. Additional conjunctival manifestations may result from antileukemic drugs, such as methotrexate-induced keratoconjunctivitis, or from opportunistic infections favored by immunosuppression [13,53]. Chemotherapy, particularly Cytarabine, can cause corneal toxicity by disrupting epithelial DNA synthesis. In GVHD, keratoconjunctivitis sicca is common and may progress to keratitis, ulcers, opacification, or corneal calcification. Immunosuppression further predisposes to infectious keratitis, which can lead to thinning, ulceration, or perforation [13]. Extramedullary relapse may present with hypopyon uveitis, while anemia or hyperviscosity can induce anterior segment ischemia with edema, inflammation, hypertension, pain, and visual loss, and cataracts may develop from treatment or ischemia [13,48]. Opportunistic infectious scleritis may sometimes develop in immunocompromised patients [13].

#### 3.1.5. Orbit and Ocular Adnexa

##### Primary Involvement

In ALL, infiltration of lacrimal glands, eyelids, orbital soft tissues, and extraocular muscles can cause exophthalmos or diplopia; the presentation mirrors other orbital masses and diagnosis requires biopsy. Eyelid involvement may manifest with edema, inflammation, chemosis, and pain [13]. Myeloid sarcoma is a rare extramedullary tumor occurring in 2.5–9.1% of AML, more frequent in children; it may precede, coincide with, or follow systemic disease, including post-transplant relapse. Imaging is nonspecific and lesions may be poorly differentiated; systemic disease often develops within one year. Proptosis is most common, with ptosis, eyelid swelling, diplopia, or vision loss also reported; lesions are usually unilateral and superior or superotemporal, whereas bilateral disease strongly suggests myeloid sarcoma [2]. The prognostic impact is debated [54,55,56,57]; chemotherapy is the mainstay while radiotherapy and early chemotherapy for isolated orbital chloroma show no prognostic benefit [2].

##### Secondary Involvement

**Figure 4:**
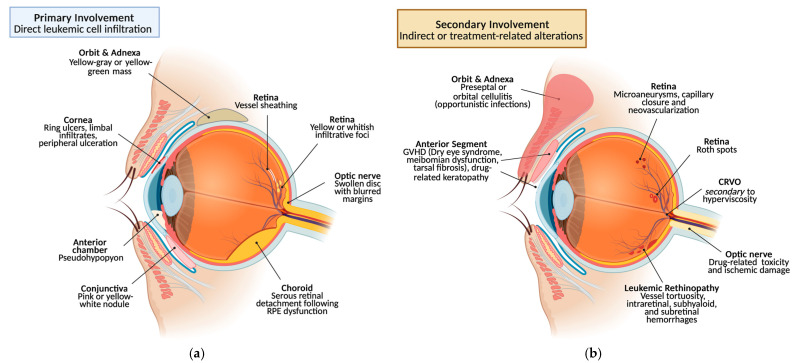
Ocular involvement in leukemia: (**a**) primary (direct) infiltration and (**b**) secondary (indirect or treatment-related) alterations [60].

Orbital involvement may arise post-remission or because of therapy or GVHD. Immunocompromised patients are prone to opportunistic infections. Lacrimal gland infiltration by leukemia, GVHD, or radiation commonly causes severe dry eye with potential corneal complications [49,58]. Infectious processes such as cellulitis or dacryocystitis require prompt antibiotic and chemotherapy management [59] (Figure 4).

### 3.2. Ocular Immune Privilege and Leukemia-Related Vascular Involvement

The eye, historically regarded as an immune-privileged organ, preserves this status through local mechanisms that actively suppress inflammation, including reduced MHC expression, anti-inflammatory cytokine release, and T-cell regulation. Immune privilege does not abolish immunity, but establishes a tightly regulated niche that safeguards ocular tissues and supports recovery after injury. Specialized vascular barriers, the blood–aqueous and blood–retinal barriers, preserve ocular homeostasis and antigen isolation but may be disrupted by inflammation or vascular occlusion, facilitating leukemic infiltration [61,62,63]. OCTA studies in acute leukemia show reduced retinal vessel density and capillary dilation, partially reversible after remission, suggesting a link between retinal microcirculation, cytopenia, and bone marrow function. Capillary dilation and disruption of the normal branching architecture in acute leukemia may reflect anemia, decreased perfusion pressure, blast-derived cytokines, or their combined effects [23]. Moreover, evidence suggests active interactions between circulating leukemic cells and vascular endothelium across multiple organs, contributing to local disease invasion even within protected niches [64].

### 3.3. Multiple Signs Association

In clinical practice, ophthalmic presentation may be more complex than the involvement of a single anatomical structure, as primary ocular involvement can coexist with secondary manifestations. This pattern is mainly observed in children, either at onset or relapse, and can present with complex findings such as conjunctival, uveal, choroidal, and orbital infiltration, often associated with proptosis, subconjunctival hemorrhage, or corneal complications. Reported cases include children with multifocal infiltration without systemic disease [65], bilateral orbital and choroidal lesions with proptosis [66,67], and retinal detachment with secondary glaucoma signaling relapse after remission [68].

### 3.4. Laterality of Ocular Involvement

In a recent comparative cohort of 244 patients with leukemic ophthalmopathy, ocular involvement at presentation was unilateral in 45% and bilateral in 55%, across all subtypes (ALL, AML, CML, CLL). This distribution supports that direct leukemic infiltration may present unilaterally, whereas bilateral disease remains common overall due to systemic vascular and choroidal involvement. Patterns also differed by subtype, with myeloid leukemias showing more hemorrhagic posterior findings and lymphoid leukemias more non-hemorrhagic anterior or posterior changes [9].

### 3.5. Ocular Signs and Prognosis

Ocular involvement in leukemia, and particularly posterior segment infiltration (excluding leukemic retinopathy), is associated with poorer prognosis and a higher risk of bone marrow relapse and CNS involvement [66,69]. Indeed, more than 50% of patients with intraocular leukemia develop CNS involvement, with even higher rates observed in those with posterior segment infiltrates [27].

Leukemic retinopathy is associated with more aggressive disease and poorer outcomes, with survival significantly reduced in patients showing retinal lesions, particularly cotton wool spots [6,70,71]. In pediatric AML and ALL, ocular or orbital involvement correlates with higher rates of bone marrow relapse and CNS infiltration, underscoring its prognostic relevance and impact on overall survival [72].

## 4. Discussion

Leukemic ocular involvement is a well-recognized manifestation of the disease, reported with variable frequency in both adult and pediatric populations. Virtually any ocular structure may be affected, either through direct leukemic infiltration or secondary complications. Importantly, ocular manifestations may occur as the initial presenting sign of leukemia or emerge during relapse, and in both settings they have been consistently associated with more aggressive disease and poorer prognosis.

To the best of our knowledge, this is one of the few reported adult cases showing simultaneous orbital, adnexal, choroidal, and retinal involvement, accompanied by hemorrhagic changes in both anterior and posterior segments. This multifocal presentation, involving several ocular structures concurrently, is highly suggestive of leukemic relapse and reflects extensive ocular dissemination rather than isolated infiltration. Previous reports have predominantly described similar concomitant ocular signs in pediatric patients [65,66,67], whereas adult presentations remain exceptionally rare. Chawla et al. [73] reported a 24-year-old patient with ALL presenting with bilateral blindness due to multiple simultaneous ocular abnormalities, including sclerokeratitis, retinal hemorrhages, and optic nerve thickening. Together with our case, these findings underscore the potential severity and complexity of leukemic ocular involvement beyond childhood.

Several mechanisms may have contributed to the ocular presentation observed in our case. Endothelial injury during the leukocytosis phase may have disrupted the vascular barriers, facilitating leukemic cell migration and localization within ocular tissues. Once sequestered in this sanctuary site, these cells may have been less responsive to systemic chemotherapy. Furthermore, the pancytopenia that followed intensive treatment may have exacerbated vascular fragility and ischemic damage, thereby aggravating the clinical presentation and contributing to the overlap of direct leukemic infiltration with secondary, treatment-related ocular alterations [23,64].

Establishing a diagnosis that can effectively guide therapy is particularly challenging when direct and indirect signs overlap. Systemic disease severity, the toxic effects of chemotherapy, and profound immunosuppression may mask or mimic ocular manifestations [49], while biopsy, often the gold standard, may be precluded due to severe thrombocytopenia and bleeding risk. In this setting, careful ophthalmic examination combined with imaging modalities such as B-scan ultrasonography and MRI plays a pivotal role in raising clinical suspicion and supporting timely recognition of relapse.

The prognostic implications of ocular involvement are considerable. Posterior segment infiltration, in particular, has been linked to higher rates of bone marrow relapse and CNS disease [27,66,69]. Leukemic retinopathy and retinal infiltration have also been correlated with more aggressive disease, reduced survival, and worse outcomes in both adult and pediatric cohorts [6,71,72]. Thus, the detection of simultaneous and multifocal ocular signs should prompt urgent systemic reassessment and therapeutic re-evaluation. It should be noted, however, that most studies on ocular involvement and prognosis in leukemia are retrospective and involve small, heterogeneous cohorts, limiting comparability and generalizability. Thus, while ocular findings seem to correlate with poorer outcomes, these associations should be interpreted with caution until confirmed by larger prospective studies.

In conclusion, this case emphasizes the importance of early recognition of ocular manifestations as a potential sign of systemic relapse. A multidisciplinary approach involving ophthalmologists, hematologists, and oncologists is essential for prompt diagnosis and treatment planning. Ocular involvement, particularly when multifocal, should be regarded as a red flag for faster disease progression, and vigilance is warranted even in patients apparently in hematologic remission.

## Data Availability

No new data were created or analyzed in this study.
